# Vaccine Hesitancy in Taiwan: Temporal, Multilayer Network Study of Echo Chambers Shaped by Influential Users

**DOI:** 10.2196/55104

**Published:** 2024-08-09

**Authors:** Jason Dean-Chen Yin

**Affiliations:** 1 School of Public Health Li Ka Shing Faculty of Medicine University of Hong Kong Hong Kong China (Hong Kong)

**Keywords:** network analysis, infodemiology, vaccine hesitancy, Taiwan, multiplex network, echo chambers, influential users, information dissemination, health communication, Taiwanese data set, multilayer network model, vaccine hesitant, antivaccination, infoveillance, disease surveillance, public health

## Abstract

**Background:**

Vaccine hesitancy is a growing global health threat that is increasingly studied through the monitoring and analysis of social media platforms. One understudied area is the impact of echo chambers and influential users on disseminating vaccine information in social networks. Assessing the temporal development of echo chambers and the influence of key users on their growth provides valuable insights into effective communication strategies to prevent increases in vaccine hesitancy. This also aligns with the World Health Organization’s (WHO) infodemiology research agenda, which aims to propose new methods for social listening.

**Objective:**

Using data from a Taiwanese forum, this study aims to examine how engagement patterns of influential users, both within and across different COVID-19 stances, contribute to the formation of echo chambers over time.

**Methods:**

Data for this study come from a Taiwanese forum called PTT. All vaccine-related posts on the “Gossiping” subforum were scraped from January 2021 to December 2022 using the keyword “vaccine.” A multilayer network model was constructed to assess the existence of echo chambers. Each layer represents either provaccination, vaccine hesitant, or antivaccination posts based on specific criteria. Layer-level metrics, such as average diversity and Spearman rank correlations, were used to measure chambering. To understand the behavior of influential users—or key nodes—in the network, the activity of high-diversity and hardliner nodes was analyzed.

**Results:**

Overall, the provaccination and antivaccination layers are strongly polarized. This trend is temporal and becomes more apparent after November 2021. Diverse nodes primarily participate in discussions related to provaccination topics, both receiving comments and contributing to them. Interactions with the antivaccination layer are comparatively minimal, likely due to its smaller size, suggesting that the forum is a “healthy community.” Overall, diverse nodes exhibit cross-cutting engagement. By contrast, hardliners in the vaccine hesitant and antivaccination layers are more active in commenting within their own communities. This trend is temporal, showing an increase during the Omicron outbreak. Hardliner activity potentially reinforces their stances over time. Thus, there are opposing forces of chambering and cross-cutting.

**Conclusions:**

Efforts should be made to moderate hardliner and influential nodes in the antivaccination layer and to support provaccination users engaged in cross-cutting exchanges. There are several limitations to this study. One is the bias of the platform used, and another is the lack of a comprehensive definition of “influence.” To address these issues, comparative studies across different platforms can be conducted, and various metrics of influence should be explored. Additionally, examining the impact of influential users on network structure and chambering through network simulations and regression analysis provides more robust insights. The study also lacks an explanation for the reasons behind chambering trends. Conducting content analysis can help to understand the nature of engagement and inform interventions to address echo chambers. These approaches align with and further the WHO infodemic research agenda.

## Introduction

### Vaccine Hesitancy and Infodemiology

Vaccine hesitancy, as defined by the World Health Organization’s (WHO) Strategic Advisory Group of Experts, refers to the reluctance or refusal to vaccinate despite the availability of vaccinations. While straightforward in definition, vaccine hesitancy encompasses a spectrum of attitudes [1]. This spectrum arises from complex interactions involving health attitudes, decision-making processes, cultural contexts, and health infrastructure. These factors contribute to varying degrees of vaccine confidence, vaccine complacency, and accessibility to vaccination services [2]. Theories borrowed from the health behavior literature underscore the complexity of vaccination intentions. For instance, the Health Belief Model posits that individuals assess the perceived severity of a disease (perceived susceptibility and severity) alongside the perceived benefits and barriers of treatment before making a decision [3]. These theories emphasize that the decision to vaccinate is influenced by a multifaceted interplay of factors. The implications of vaccine hesitancy are significant, as evidenced by the resurgence of diseases such as measles, with studies showing that even a small drop in vaccination rates can lead to substantial increases in disease outbreaks and cost health care systems millions [4,5]. Consequently, the WHO identified vaccine hesitancy as one of the top 10 global health threats in 2019 [6].

Implicit in the definition of vaccine hesitancy is its dynamic nature across a spectrum, indicating that attitudes toward vaccination can evolve over time. Monitoring this phenomenon necessitates a flexible, dynamic approach to complement traditional epidemiological methods. Social media has become increasingly utilized for this purpose due to its widespread adoption and the real-time nature of the data it offers. This enables prompt detection of changes in public sentiment. Furthermore, social media plays an increasingly influential role in shaping public perceptions of vaccination. This influence was particularly pronounced during the COVID-19 pandemic, where misinformation proliferated alongside scientific communication, complicating public health responses [7,8]. The pandemic brought renewed focus on vaccine hesitancy studies, especially with the development and scrutiny of COVID-19 vaccines. This prompted the WHO to increase investment in infodemiology research.

The WHO outlined a comprehensive research agenda for managing infodemics, which represents a situation in which an excess of information, including false or misleading content, spreads in both digital and physical spaces during outbreaks [9,10]. This framework is organized into five 5 streams, each targeting various aspects of infodemic management. The first stream focuses on measuring and monitoring the evolution of infodemics, utilizing various metrics and tools to track information flow. A subtheme within this stream involves triangulating data from multiple sources and standardizing taxonomies and classifications. The second stream aims to detect the origin, evolution, and spread of information across various platforms, with a specific emphasis on the influence of key actors in disseminating this information. The third and fourth streams focus on deploying and evaluating interventions that mitigate the effects of infodemics, thereby enhancing community resilience. The overarching goal of the research agenda, encapsulated in the final stream, is to integrate these tools into broader epidemic management strategies, promoting a paradigm shift in epidemic management that incorporates infodemic control.

In the vaccine hesitancy infodemic space, the majority of studies have primarily concentrated on the first and second streams. A review by Yin [11] provides a summary of the most commonly used big data methods and key research topics in vaccine hesitancy. However, several gaps remain in this area. One significant gap is the need for more comprehensive tracking of vaccine sentiment. While many studies focus on identifying either pro- or anti-vaccination sentiment [11,12], there is a notable lack of attention given to the gray area of vaccine “hesitancy,” which represents a potentially vast spectrum. Another gap is the predominance of studies focusing on English, possibly influenced by its global linguistic dominance as a *lingua franca* and the availability of tools for English-language analysis. Analyzing diverse contexts and languages enriches the vaccine hesitancy discourse by triangulating global trends with context-specific insights. A third gap lies in the exploration of thematic areas that remain underexplored. Many studies use sentiment analysis and topic modeling as relatively straightforward tools for tracking vaccine sentiment. However, these methods have limitations in identifying how sentiments cluster and the key actors responsible for such clustering. Another frequently neglected thematic area is the temporal aspect of sentiment change. Given that vaccine hesitancy is a dynamic state, tracking these changes over time is crucial for comprehensive understanding. For these reasons, this study focuses on vaccine hesitancy in Taiwan during the COVID-19 pandemic using social network analysis. The choice of methods, the case study of Taiwan, and the emphasis on influencers aim to contribute to the WHO’s infodemiology efforts by proposing new surveillance methods, providing insights from a distinct context for triangulating broader trends, and exploring the understudied area of vaccine hesitancy (influencing). The following sections delve into the literature on echo chambers and influential users in vaccine hesitancy, offering conceptual clarifications, a task aligned with the WHO’s infodemic research agenda.

### Echo Chambers, Influential Users, and Information Transmission

#### Overview

Social networks exert a growing influence on decision-making processes. Grounded in Social Network Theory, individuals leverage personal networks to access relevant information and support from peers [13]. These networks enable individuals to gather social cues from others with similar experiences, fostering a sense of belonging and aiding in identity formation [14-16]. As a result, integrating health communication into these contexts can potentially facilitate behavior change across various health issues. This change is facilitated by modifying the mediators of health decision-making, particularly by reinforcing perceived efficacy and self-efficacy through social networks [15]. Consequently, social networks are well-suited for health promotion, leveraging their mass scale and interpersonal properties.

While social networks foster a sense of identity and belonging, this characteristic can also pose a potential pitfall. Homophilous communities, also known as echo chambers, perpetuate and reinforce specific ideas or beliefs, potentially distorting perception and normalizing ideologies that diverge from the mainstream. This polarization solidifies individuals in their own viewpoints, restricting exposure to diverse perspectives [17-19]. In public health, when these entrenched views contradict best practice recommendations, the implications for health behavior and outcomes can be substantial. This phenomenon is particularly evident in the context of vaccine hesitancy [20], where polarization and echo chambers have fueled the persistence and expansion of hesitant narratives, thereby impeding vaccination efforts. It is crucial to identify when and by whom this occurs to effectively guide health communication strategies aimed at mitigating these effects.

#### Echo Chambers

Echo chambers have garnered renewed attention, largely due to significant shifts in technology, media, and communication over the past decade. Originally a metaphor from acoustic environments where sounds reverberate in enclosed spaces, “echo chambers” now refer to the phenomenon of amplifying and reinforcing ideologies within closed, like-minded communities.

The phenomenon can largely be broken down into 2 processes: chambering and echoing. Chambering occurs as individuals naturally segregate into groups with like-minded preferences, beliefs, and attitudes. Echoing ensues when those within the chamber influence others in a nonrational manner with their beliefs. These processes are interconnected and often coevolve, sometimes sequentially. For instance, many users primarily seek out content that is relevant or interesting to them [21]. This behavior is amplified by social media algorithms that prioritize content similar to what users have previously engaged with [22]. Consequently, users are more likely to connect with others who share similar tastes and preferences. Additionally, individuals may actively avoid information that contradicts their worldview [23]. Chambering, where individuals segregate based on shared beliefs, is a prerequisite for the echoing effect.

In the context of vaccines and vaccine hesitancy, online echo chambers have primarily been studied to identify their existence, despite the frequent use of the term “echo chamber.” Chambers are often operationalized by demonstrating polarization or homophily to illustrate clustering. The consensus in current research leans toward confirming the presence of chambering. Several large-scale studies conducted on Twitter have indeed confirmed the presence of a chambering effect. For instance, Cossard et al [24] found that skeptics and advocates for vaccination tend to reside in separate homophilous clusters, with skeptics forming a tighter cluster and advocates distributed across several smaller clusters. Johnson et al [25] partially corroborate this finding, noting that antivaccination communities often engage more with undecided communities than with provaccination communities. Crupi et al [26], in their study on COVID-19 networks in Italy, observed that while there is convergence on certain topics of discussion, smaller communities focused on antivaccination issues, such as conspiracy theories or concerns about vaccination passports, persist. Mønsted and Lehmann [27] found similar dynamics globally on Twitter/X, where subgroups exhibit preferential attachment—a measure of homophily—resulting in what they term “epistemic echo chambers.” Moreover, this phenomenon extends to platforms of different natures. Schmidt et al [28] discovered that the consumption of vaccine-related content on Facebook is characterized by an “echo chamber effect,” with polarization intensifying over time. Van Raemdonck [29] also observed that chambering occurs differently based on platform structure. Using Facebook and Reddit as examples, they found that Facebook uses “groups” to shield users from outside ideologies, while Reddit naturally forms chambers through reinforcement against external challenges. Meyer et al [30] found that polarization also exists on web forums for the Canadian Broadcasting Corporation, particularly in debates surrounding flu vaccinations.

Less researched is the association between chambering and echoing, particularly in the context of vaccine hesitancy. However, only a few studies have explored this because of the challenges in linking online behavior to beliefs and actions. Research in this area has typically relied on survey data, modeling, or experimental methods. For instance, Jennings et al [31] found that individuals who primarily rely on platforms such as YouTube for information are more likely to encounter misinformation, believe in conspiracies, and exhibit a lower willingness to vaccinate. This study, however, did not directly measure online echo chambering but rather indirectly assessed it through a questionnaire that identifies users who engage with a limited subset of online media, linking this behavior to beliefs. In modeling approaches, Müller et al [32] investigated how the emergence of echo chambers around measles contributed to the occurrence and persistence of antivaccination opinions, suggesting a significant chambering effect. Phillips and Bauch [33] argued that echo chambers serve as early warning signs of vaccine hesitancy and can thereby influence infection dynamics. The study by Giese et al [34] on flu found that individuals tend to find incoming information more convincing if it aligns with their existing attitudes.

Although measuring echoing, particularly its direct connection to behaviors or beliefs, is challenging, its association with chambering makes the latter a suitable starting point to analyze potential impacts on behavior (ie, true “echo chambering”). Measuring chambering across different platforms and contexts contributes to this body of knowledge, which is a primary aim of this study. Additionally, the evolution and changes over time in these dynamics are understudied but essential for understanding temporal intervention points. Lastly, in addition to measuring the macroscopic phenomenon of chambering, understanding the individuals who contribute to this phenomenon sheds light on the echo chambering process. This understanding is crucial for targeted promotion efforts aimed at vaccination.

#### Influential Users

Katz’s [35] 2-step flow of communication hypothesis, proposed in 1957, suggests that mass media messages reach the public through the mediating role of opinion leaders [6]. (Often, there is a distinction between opinion leaders [sometimes referred to as key opinion leaders] and influencers. This manuscript uses the words interchangeably.) These opinion leaders, often characterized by qualities that resonate with a group’s interests or circumstances, wield significant influence over group opinions on relevant issues [7]. Before the internet, professional groups such as doctors and nurses commonly played the role of opinion leaders in health, enjoying significant trust from their patients. Nonprofessionals, such as individuals in high schools, could also serve as opinion leaders influencing their peers’ health decisions [8,9]. In both scenarios, trust was a crucial trait for exerting influence. In the social media era, influence is decentralized, giving rise to online influencers.

Identifying influencers and understanding the extent of their influence are crucial for research in this area. Influencers can be identified through their real-world identity (eg, the US Centers for Disease Control and Prevention [CDC] on platforms such as Twitter/X as a health influencer). They can also be recognized by their potential for message diffusion [36], their opinions and stances on topics, or their tone (eg, individuals with high message dissemination potential or those advocating for vaccination being provaccination). Network measures such as centrality or community detection, known as topological measures, can also be used (eg, connectedness within a network as a proxy for influence). To comprehend their sphere of influence and potential reach, Bamakan et al [37] offer a valuable framework. In their review of marketing influencers, they propose 4 aspects through which opinion leaders can exert influence [7]. One aspect is breadth, indicating their local or global influence over domestic or international markets. The second aspect is the diversity of topics, indicating their degree of specialization, ranging from single-topic specialists to diverse, multitopic experts. The third aspect is polarity, whether they promote positive or destructive messaging (eg, antivaccination). The fourth aspect is temporality, indicating their short- or long-term influence on their community. This framework will be crucial in delineating the scope of the overall study.

In health literature, online influencers are frequently leveraged to promote evidence-based public health behaviors and accelerate the diffusion of health innovations and promotions [10,11]. Regarding vaccines, studies have explored the role of online influencers in both promoting and discouraging vaccination. For instance, in the context of the human papillomavirus vaccine, “mommy bloggers” with trusted reputations in new-mother forums successfully promoted human papillomavirus vaccination [5,12]. Online medical expert celebrities, utilizing increased interactivity and promotional content, successfully promoted COVID-19 vaccination in China [21]. However, these promotional efforts were often countered by the actions of other types of influencers. Whereas health experts promoted vaccination, politicians used their platforms to express dissent, fostering distrust and posing threats to vaccination campaigns [22,23]. Analyzing who the influencers are and understanding their role in the network is crucial for the dual effort of increasing vaccine promotion messages and reducing antivaccination content.

Less studied, however, is the role of influencers in driving the chambering effect. While chambers represent a macro-network phenomenon, they are fueled by individual users—micro-network contributors—whose activity within the network may contribute to chamber formation. These users’ connectivity serves as an indicator of their engagement within the overall network, also acting as a proxy for their ability to disseminate information. The capacity to spread messages is crucial due to its implications on the nature and effectiveness of message dissemination. For proponents of vaccines, wide-reaching messages are essential to promote a provaccination stance. Conversely, within antivaccination communities, messages that fail to spread circulate among insulated groups, reinforcing the antivaccination sentiment. Focusing on the upstream factor of opinion leaders’ role in echo chamber formation helps understand their ability to catalyze or deter vaccination efforts.

However, studying the influencer’s role in driving echo chambers requires clarity on its scope. Although measuring influence on behavior is challenging in an infodemic study, examining communication dynamics within a network to understand chambering is more feasible. Additionally, using the 4 aspects mentioned—locality, diversity, polarity, and temporality—can aid in refining measures and delineating the study’s scope. This study investigates the formation of chambers within a local forum regarding varying stances on vaccines over time. Initially, the study takes a macroscopic view and then shifts focus to examine the role of influencers. By concentrating on the upstream factor of chambering, the study aims to evaluate its potential impact on promoting or discouraging vaccination efforts.

This study utilizes data from a Taiwanese forum to investigate the following question: How do engagement patterns within and across COVID-19 stances contribute to the formation of chambers over time? This exploration begins by examining the layer level (macro-network), followed by an analysis at the node level (micro-network). At the macro level, the study explores pairwise connectivity between layers over time. At the micro level, it identifies highly connected nodes (opinion leaders) and evaluates their engagement behavior within the multilayer network to assess their role in chambering.

## Methods

### Data and Approach

PTT is a terminal-based bulletin board system (BBS) in Taiwan developed by Yi-Chin Tu (杜奕瑾) and other students from National Taiwan University in 1995 [38]. It serves as a free and open forum where users can discuss a wide range of topics including politics, culture, entertainment, and current affairs. Often referred to as Taiwan’s “Reddit,” PTT is one of the most active forums in Taiwan. From July 2022 to July 2023, the average number of users per day was 56,000 [39]. The user base includes adolescents to young adults, predominantly male, and the forum can host politically charged and radical opinions, similar to Reddit.

The empirical strategy involves evaluating chambering at the network level, identifying opinion leaders based on their network connectivity, and documenting how their behavior influences chambering dynamics over time.

I argue initially that the locality and diversity—the first 2 aspects of influencers—are confined to the forum and its topics. Extracting all vaccine-related discussion boards on PTT focuses the discussion on a case study specific to Taiwan. Next, to analyze polarity, I categorize these boards into 3 sentiments—provaccination, vaccine hesitant, and antivaccination—within vaccine-related discussions. These stances form a conceptual multilayer network, where each layer represents a sentiment toward vaccination. Subsequently, various layer-level analyses are conducted both at the macro and micro levels over time, taking into account the fourth aspect, temporality, for influencers.

### Network Representation

#### Structure of PTT

**Figure 1:**
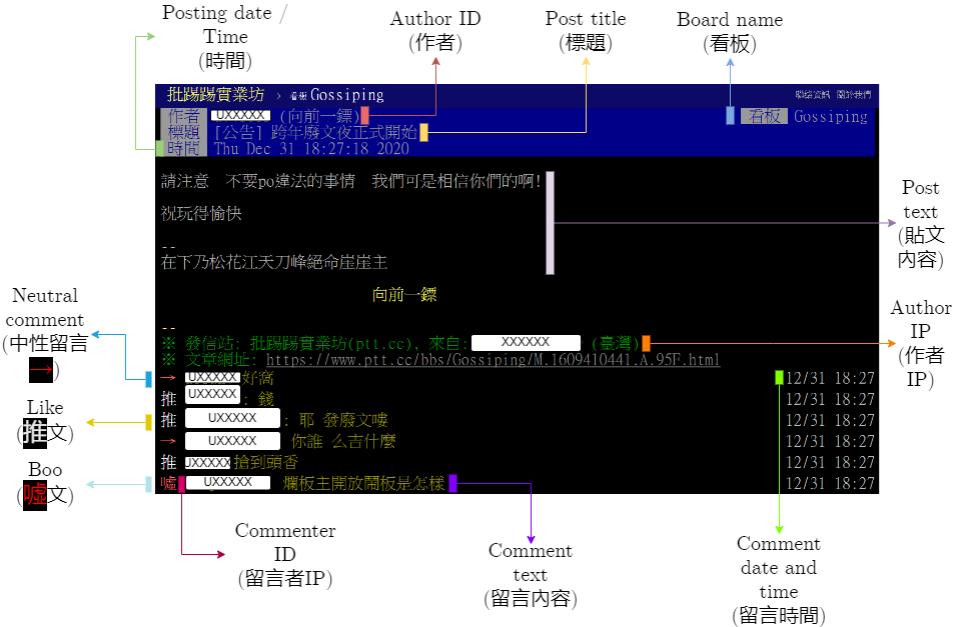
Chinese and English labelling of 1 thread of the PTT forum to show its structure. Usernames omitted for protecting individual identity.

The web-based version of PTT is structured with terminology in both English and Chinese. The bulletin consists of boards (看板) and board masters (版主), which correspond to subject areas and administrators on any forum. Posts within each board can be made in 2 ways: by creating a new post (PO文 or 貼文) or by replying to a post (回應文). Within each post, users have the option to leave a follow-up comment that expresses sentiment: they can like (推), boo (嘘) (equivalent to a “hiss” or “shh” sound), or leave a neutral reaction (→). These reactions are denoted by corresponding Chinese characters in the comment section, or simply an arrow for neutral reactions. Comments are limited to 39 characters per line. Each post includes user information such as posting time (時間), author ID (作者), title (標題), and IP address (作者IP). Additionally, information on commenters’ posting dates, times, and IPs is provided. All of these are captured in Figure 1.

The raw corpus comprises all posts from the “Gossiping” board on PTT spanning from January 1, 2021, to December 1, 2022. “Gossiping” is the largest and most active board on PTT, covering a wide range of topics. The start date coincides broadly with the initiation of a government vaccination campaign, while the end date marks Taiwan’s reopening to tourists. This time frame captures significant vaccine-related events in Taiwan, including vaccine procurement, outbreaks, domestic vaccine development, discovery of COVID-19 variants, and changes in vaccination policy, among others. All posts were collected using a web-crawler (ptt-web-crawler) developed by GitHub user “jwlin.”

To identify vaccine-related message boards, the filter term “vaccine” (疫苗) was used to search through the raw corpus and extract relevant boards. Each discussion board was independently labeled by 2 assessors as “provaccine,” “vaccine hesitant,” or “antivaccine” according to Table 1. These criteria have been predefined after consulting the literature on the scope and definitions of vaccine hesitancy [1,2,40], with a particular focus on efficacy appraisals as defined by the Health Belief Model [41]. It is important to note that hesitancy encompasses a broad spectrum, and the categories are designed to encompass various forms of “hesitancy,” including those who have “refused” vaccination due to lack of access or other reasons. As the filter using “vaccine” is nonspecific, we anticipated that there may be 2 irrelevant categories of classifications. The first category is “neutral,” which includes reposts of vaccine-related statistics (often related to stocks or vaccination coverage) intended without expressing an opinion on uptake. These news items are distinct from other articles that may include a stance or reason for sharing (eg, sharing news on the drawbacks of vaccination). The second category is “irrelevant” vaccination, which may tangentially mention vaccines without direct relevance to the discussion. These categories were excluded from the final network and not included in the analysis. The target interrater agreement goal was set at 85% or higher, with any discrepancies resolved by the main author. The final agreement reached 83% (4829/5818 boards), with the remaining decisions made by the main author.

**Table 1:** Provaccination, vaccine hesitant, and antivaccination board classification criteria.

COVID-19 vaccine layer	Example
Provaccination	Claim will get vaccinated once available. Discussing vaccine efficacy or safety with the intention of promoting vaccination. Announcing that one has been vaccinated. Supporting any part of the vaccine approval process. Advocating for getting vaccinated.
Vaccine hesitant—doubting efficacy	Saying they will wait and see if the vaccine is safe or effective. Doubtful or worried about the quick approval process of the vaccine. Indifferent to get vaccines as a result of perceived low risk of getting diseases. Suspicious of vaccine side effects. Mentioning that they have side effects after vaccination.
Vaccine hesitant—barriers to access	Claiming no opportunities to get vaccinated (cannot book an appointment). Discuss the excessive time and energy needed to find/book an appointment
Antivaccination	Religious or philosophical objection or refusal. The belief that vaccination interferes with natural immunity. Criticizing the vaccine industry. The belief that vaccination is dangerous and would not take it. The belief that vaccination is against human rights/is infringing on individual rights. Not choosing to vaccinate for reasons related to the government.
Neutral	Vaccine-related news with no opinion on the uptake (eg, vaccination rates, economic, insurance, stock news). Listing vaccination rates, or COVID-19 case rates. If news points in the direction of any category 1-4, put it in the corresponding category.
Irrelevant to vaccination	Discussion of other vaccines that are not related to the COVID-19 vaccine. Mentions “vaccine” but does not elaborate on opinion.

The classifications of “provaccine,” “vaccine hesitant,” and “antivaccination” represent the 3 layers (or stances) of the multilayer network, used interchangeably. To obtain network data, each row was “flattened” such that 1 row represented a directional author-commenter pair. For each row, several steps were taken. First, depending on the direction of the comment, I assigned provaccine, vaccine hesitant, or antivaccination labels based on their alignment with the sentiment of the original labeled post. For example, individuals who “like” a provaccination thread were categorized into the provaccination layer, and neutral comments were also classified as provaccination. Commenters who “boo” a thread were assigned to the opposing camp (eg, those who “boo” provaccination boards were placed in the antivaccination camp). In cases where a comment could reasonably fit into 2 categories (such as “booing” an antivaccination post, which could indicate vaccine hesitancy or provaccination), a random assignment was made. Additionally, each row was assigned a weight based on the sentiment of the comment: “likes” were assigned a weight of 2, “neutral” a weight of 1, and “boo” was weighted as 0.2. These weights were chosen to reflect varying degrees of affinity between nodes; however, they do not indicate a linear increase in weight. Negative weights were avoided in this structure due to complications in establishing shortest paths when negative weights are used in network construction.

#### Temporal Multilayer Framework

A multilayer network graph *M* consists of 3 layers *M* = {*G^P^*, *G^H^*, *G^A^*}, with each layer being a directed, weighted graph that represents the aggregate connections between commenters and authors for vaccine sentiments provaccination (*P*), vaccine hesitant (*H*), and antivaccination (*A*)*.* Each layer *l* ∈ *M* of graph *G* consists of all interactions of the set of nodes *V^l^* and set of edges *E^l^* and is represented as *G^l^* = (*V^l^*, *E^l^*), with each node being a user and each edge being a comment on that user’s post. This multilayer structure allows users to participate on multiple layers (ie, the equivalent of saying they may express various sentiments on vaccination). Constructing the network in such a way allows for the comparison across different sentiment layers on various network metrics. This is the static representation.

For the evolving network, assume that we observe the network over a finite time *T*, with starting point *t_s_*=0 and ending point *t_e_*=*T*. Each layer in *M* is defined as *G^l^*_0,_*_T_* = (*V*,*E*_0,_ *_T_*) on a time interval [0, *T*] which consists of a set of nodes or vertices *V* and a set of temporal edges *E*_0,_ *_T_*. The evolving multilayer network is thus *M*_0,_ *_T_* = {*G^P^*_0,_ *_T_*, *G^H^*_0,_ *_T_*, *G^A^*_0,_ *_T_*}. This multilayer, temporal network is observed at discrete time points *t*_1,_ *t*_2_,..., *t_n_*_–1_, *t_n_*. At any time point *t_n_*, an instantiation of the multilayer, *M_n_*, is observed, whereby each *G^l^_n_* contains the set of temporal edges *E^l^_n_* such that 
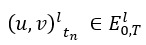
 with edges between nodes *u*, *v* contained within the period 
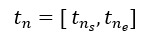
 such that 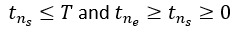 (ie, the instantiation time is between the start time *t_s_* and end time *t_e_*, and the end time *t_e_* is later than the start *t_s_*). The graphs, being directed, are also nonmirrored such that (*u*, *v*) ≠ (*v*, *u*).

### Statistical Analysis

#### Degree Growth

Influential accounts are those that are very interactive in or across networks. They generally have 2 characteristics: they receive many comments from different users (opinion leaders) and give many comments to different users (engagers). We measure the first characteristic as *indegree* and the second as *outdegree*. Together, the 2 measures for each node indicate an account’s activity, and consequently its influence within the network. In addition, as an extension, because the discussion network is multilayer, we use 3 new metrics from Nguyen et al [42] to account for cross-layer interactions, termed *cross-layer metrics*. Correspondingly, we have *cross-indegree* and *cross-outdegree* [42]. These are derivatives from the larger literature of cross-layer measures for multiplex networks [43].

#### Indegree

The indegree *d^l^_in_* of node *v* in layer *l* measures the number of edges pointing inward to *v*. If the number of in-neighbors of node *v* in layer *l* is *N^l^_in_*(*v*) = {(*u*, *v*) ∈ *E^l^*}, where *E^l^* is the set of edges and *u* is any other neighbor, the indegree is calculated as *d^l^_in_* (*v*) = |*N^l^_in_* (*v*)|. Any account with a high indegree means that the user has received a lot of attention from other users in the same layer *l*.

#### Diversity: Cross-Indegree

The cross-indegree *d^M^_in_* of node *v* in the multilayer network *M* measures the number of edges pointing inward to *v* across all layers. If the number of unique in-neighbors across the different layers of the network for node *v* in multilayer network *M* is the union across layers 
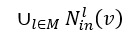
, the cross-indegree is calculated as 
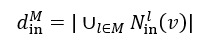
 (this calculation is the same as counting the number of unique in-neighbors across the entire network). The higher the cross-indegree, the more that an author attracts attention from users in other layers, or across the network (high engagement across the 3 vaccine sentiments).

#### Outdegree

The outdegree *d^l^*_out_ of node *v* in layer *l* measures the number of edges pointing outward from *v*. If the number of in-neighbors of node *v* in layer *l* is *N^l^*_out_(*v*) = {(*v*,*u*) ∈ *E^l^*}, where *E^l^* is the set of edges and *u* is any other neighbor, the outdegree is calculated as *d^l^*_out_(*v*) = |*N^l^*_out_(*v*)|. Any account with a high outdegree means that the user has commented on many other users in the same layer *l*.

#### Diversity: Cross-Outdegree

The cross-indegree *d^M^*_out_ of node *v* in the multilayer network *M* measures the number of edges pointing outward of *v* across all layers. If the number of unique out-neighbors across the different layers of the network for node *v* in multilayer network *M* is the union across layers 
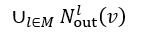
, the cross-outdegree is calculated as 
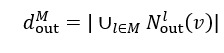
. The higher the cross-outdegree, the more that an author engages from users in other layers, or across the network per similar reasoning (indicating commenting across the 3 vaccine sentiments).

### Engagement Growth

#### Spearman Rank

The activity of certain nodes differs across different layers in the multilayer network. To verify whether the active users differ across different layers of the network, I conducted a Spearman rank correlation to measure the similarity between nodes in common for each pairwise combination of layers. This is done for indegree and outdegree because it is a directed network, and measures different aspects of nodes in a network. Correlations run between –1 and 1, with the former indicating a negative correlation between ranks and the latter indicating a positive correlation. Spearman ranks were conducted at each time *t_n_* for temporal visualization, with the total at time *T* representing the entire Spearman across the entire network. A descriptive metric of the number of overlapping nodes in each pairwise layer was also done to see how nodes generally participate across sentiment layers, both in total and over time.

#### Temporal Diversity (Macro)

To understand temporal interactions between layers, 2 different levels of analysis were performed. One is the macroscopic network level, assessing how average cross-indegree and cross-outdegree change over time for layers. The second is at the microscopic network level, focusing on how nodes in each layer interact across the 3 layers, and how this changes over time.

For the macro-level, the cross-indegree of a node *v* in the multilayer network *M*, *d^M^_in_*, is the union of inward edges across the entire network. Similarly, the cross-outdegree of a node *v* in the multilayer network *M*, *d^M^_out_*, is the union of edges pointing outward from *v*, and is a measure of commenting activity into the multilayer network. These 2 equations account for the number of comments received and given, respectively, for each *node* for each layer. To aggregate either measure up to the layer level *l*, we sum these values across all nodes in the layer to get the layer cross-indegree, *l^M^_in_*, and layer cross-outdegree, *l^M^_out_*:


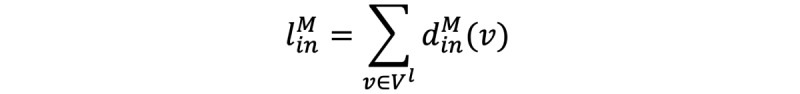



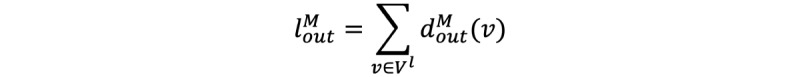


where *v* ∈ *V^l^* indicates all possible nodes in the given layer, and then averaged across all nodes in the layer. The values are computed at each time point *t_n_* and plotted over the interval [0, *T*] to see how receiving and commenting activities happen temporally. An overall estimate was also made of the multilayer network by taking the value at time *T*, assuming the network at that time represents the entire network of connections until the right censoring time.

#### Temporal Degrees (Micro)

For the micro level, I looked at how select nodes (influential nodes) in each layer branched across the 3 layers over time. This was done for 2 types of users: highly diverse nodes and hardliners. The results of these gave an idea of how much different node types contribute to the echo chambering effect. The former compares the participation sites (ie, layers) of nodes that are highly engaging on the forum to see where they are engaging. The latter compares the participation of individuals that only exist within their layer to drive conversation within their layer, entrenching the echo chamber. For highly diverse nodes, the top 50 nodes were taken and their indegrees and outdegrees were measured across each layer for comparison.

For the latter, an entropy constant was used to determine the distribution of connections across the 3 layers. For each node *v*, *p*(*l*)*_v_* was calculated, which is the probability of a connection extending from node *v* being in layer *l*. For this purpose, the number of connections any node *v* has in a layer *l* is divided by the node in all other layers. The entropy for that node in that layer, *H_v,l_*, is then computed as follows:


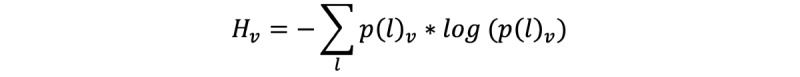


with higher values indicating more equal distribution. To identify the hardliners, the 30 lowest values of entropy within each layer were identified, and their indegree and outdegrees were compared in each layer (across layers).

#### Overlapping Nodes

A descriptive metric of the number of overlapping nodes in each pairwise layer was done to see how nodes generally participate across sentiment layers, both in total and over time.

#### Validation: Bootstrapping

Layer classification was performed by 2 independent raters following predefined criteria for stance identification. Although these criteria were developed based on existing literature, there remains a possibility that classifications and resulting statistics could occur by chance. To assess this variability, I conducted all measurements using averages derived from a bootstrap sample (n=1000) as a benchmark for statistical estimates.

### Ethical Considerations

All data from PTT are open and publicly available. All data from PTT in its raw form include usernames.These usernames are deidentified and anonymised during the research process to ensure they cannot be traced back to individuals. To do this, a 6 digit alphanumeric temporary username is generated for each unique user. Data is available upon request.

## Results

### Network Description

From a total of 48,288 scraped boards, 2992 were selected for analysis after categorization: 1283 were provaccination, 1322 were vaccine hesitant, and 387 were antivaccination (Table 2). Boards classified as neutral and irrelevant (n=2826) were excluded from the analysis. The number of nodes and edges in the network correspond to users and comments, respectively. The discussions on the vaccine hesitant layer are the most active with most nodes (*v*=14,037) and edges (*e*=33,520), followed by the provaccine layer (*v*=11,087, *e*=23,504). Average degrees mirrored this trend, with the vaccine hesitant layer showing the highest degrees and the antivaccination layer the lowest.

**Table 2:** Properties of the multilayer network by layer (for average degree, bootstrap n=1000).

Layer	Number of posts, n	Nodes (*v*; average)	Edges (*e*; average)	Average degree
*G^P^*	1283	11,087	23,504	4.24 (4.21-4.27)
*G^H^*	1322	14,037	33,520	4.77 (4.75-4.80)
*G^A^*	387	5477	8696	3.18 (3.15-3.20)

**Figure 2:**
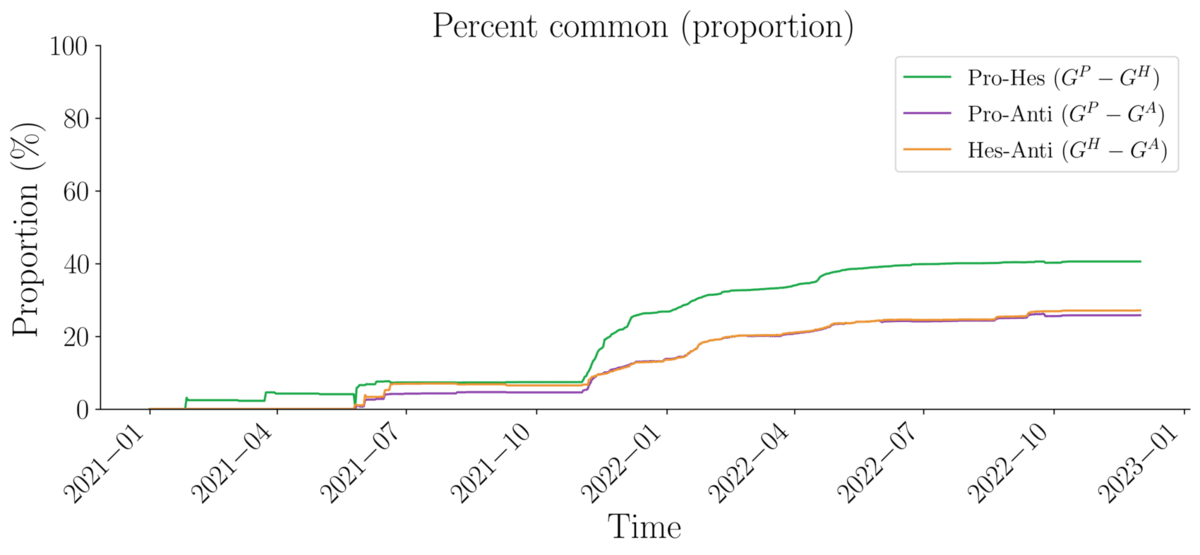
Percentage of overlapping nodes in each sentiment layer over time. anti: antivaccination; Hes: hesitant; pro: provaccination.

Based on the node overlap results across layers, it appears that a significant portion of nodes engaging in provaccination topics also participated in vaccine hesitant discussions (Figure 2). This pattern persists consistently over time, indicating ongoing engagement in both topics. A notable sharp increase is observed around November 2021, reflected in the steepening slope of the overlapping curve between provaccination and vaccine hesitant discussions. This trend could be attributed to heightened discussions following the Omicron outbreak. The overlap proportion of nodes engaging in antivaccination topics appears lower for both, indicating that these discussions occur on a smaller scale and are more isolated within the overall network.

**Figure 3:**
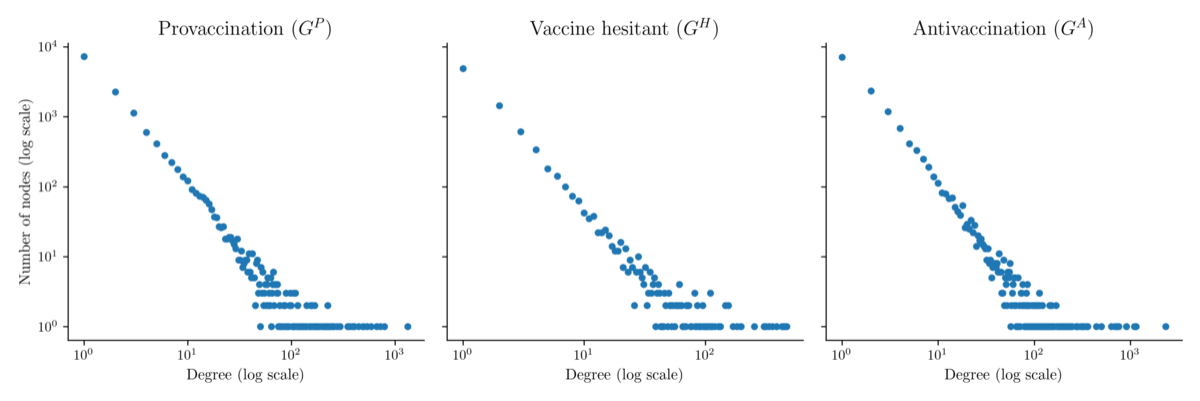
Log-log plots of number of nodes by degrees to determine power-law structure.

In network studies, a common analysis involves examining whether networks exhibit power-law distributions, characterized by a connection pattern where a few nodes have many connections and most nodes have few. Figure 3 shows histograms of node degree distributions on a logarithmic scale, visually illustrating a power-law distribution with a heavy tail. The sigma, an exponential parameter of the power-law distribution, was estimated for each layer. Layers that adhere to power-law networks exhibit probability distributions of degree *d*, *p*(*d*), following a power-law relationship *p*(*d*) *d^δ^*, where *δ*≥1 is an exponential parameter (typically with a value around 2). Upon estimating sigma for the power-law parameter, it was found that all 3 layers have sigma values of around 2. For the significance test, where significant *P* values (ie, *P*≤.05) indicate a deviation from the power-law value *δ*≈2, all layers exhibited a power-law network (*G^P^*: *δ*=2.2, *P*=.067; *G^H^*: *δ*=2.3, *P*=.068; *G^A^*: *δ*=2.3, *P*=.09). This suggests that all 3 layers are characterized by a distribution where a few nodes have many connections. A previous study on PTT also identified a similar power-law structure on the platform [42].

### Macro-Network Echo Chambering

Table 3 presents Spearman rank correlations for the entire network. It shows that, on average, the ranking based on indegree is higher than that based on outdegree. This implies that individuals who receive many comments within 1 layer also tend to do so in other layers. Particularly notable is the strong correlation between the provaccination and hesitant layers, indicating a significant overlap in nodes between these 2 layers as a partial explanation for this trend. The hesitant-antipair also shows a comparable trend, with high rankings in indegree suggesting that similar users receive comments across these layers, despite their smaller overlap in node percentage. In terms of commenting activity, overall correlations weakened, indicating that users tend to comment on sentiment boards aligned with their stance, possibly indicating an echo chamber effect. Across all pairwise comparisons of layers, the provaccination and antivaccination layers exhibited the least correlation in indegree and outdegree rankings, suggesting polarization within the network. The low overlap in node percentage further supports this observation.

**Table 3:** Pairwise Spearman ranks by indegree and outdegree metric by pairwise layer comparison (overall, bootstrap n=1000).

Layer	Indegree, mean (CI)	Outdegree, mean (CI)
(*G*^*P*^, *G*^*H*^)	0.8698 (0.8693-0.8703)	0.5831 (0.5825-0.5838)
(*G*^*P*^, *G*^*A*^)	0.5035 (0.5025-0.5044)	0.4212 (0.4200-0.4224)
(*G*^*H*^, *G*^*A*^)	0.6138 (0.6129-0.6146)	0.46130 (0.4602-0.4623)

**Figure 4:**
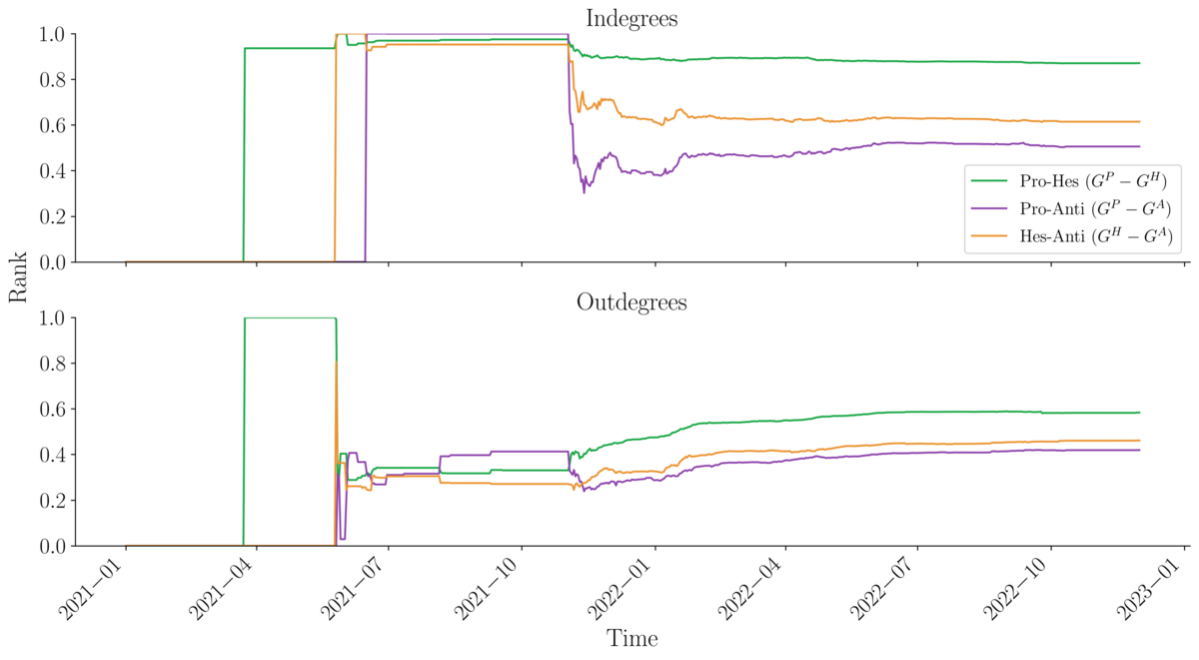
Pairwise Spearman ranks by indegree and outdegree metric by pairwise-layer comparison (temporal). anti: antivaccination; Hes: hesitant; pro: provaccincation.

These trends vary slightly over time (Figure 4). From the initial period to November 2021, we observe a high correlation in users receiving comments across different layers, indicating a small group of key users generating boards of interest. However, in terms of commenting activity, the correlation between provaccination and antivaccination sentiments is highest, suggesting some disruption in the observed echo chamber effect. Simultaneously, the overall percentage of overlapping nodes remains low (considering that Spearman ranks are compared only among similar nodes). As the network expands and the percentage of common nodes increases between the provaccination and hesitant sentiment networks, we also observe a sharp decline in users receiving and giving comments across the provaccination and antivaccination layers, suggesting a temporal polarization effect. This trend becomes particularly noticeable around November 2021.

When comparing across layers, the antivaccination layer exhibits the highest average indegree and outdegree diversity overall (Table 4). To elucidate this trend, it is important to note that while the Spearman rank only considers nodes appearing in multiple layers (indicating engagement across different vaccine sentiments), the layer average encompasses all nodes, whether they participate in multiple layers or only 1. Therefore, while common nodes may suggest some attenuation of chambering in receiving comments and polarization in giving comments, there are also unique nodes that span multiple layers. This trend is most pronounced for the antivaccination layer, indicating that it is the least chambered among the layers based on the activity of its constituent nodes, despite its relatively smaller size.

**Table 4:** Average indegree and outdegree diversity by layer (total).

Layer	Indegree diversity, mean (CI)	Outdegree diversity, mean (CI)
*G^P^*	4.62 (4.59-4.64)	4.44 (4.43-4.45)
*G^H^*	4.43 (4.41-4.48)	4.84 (4.83-4.85)
*G^A^*	6.16 (6.05-6.27)	6.95 (6.89-7.00)

**Figure 5:**
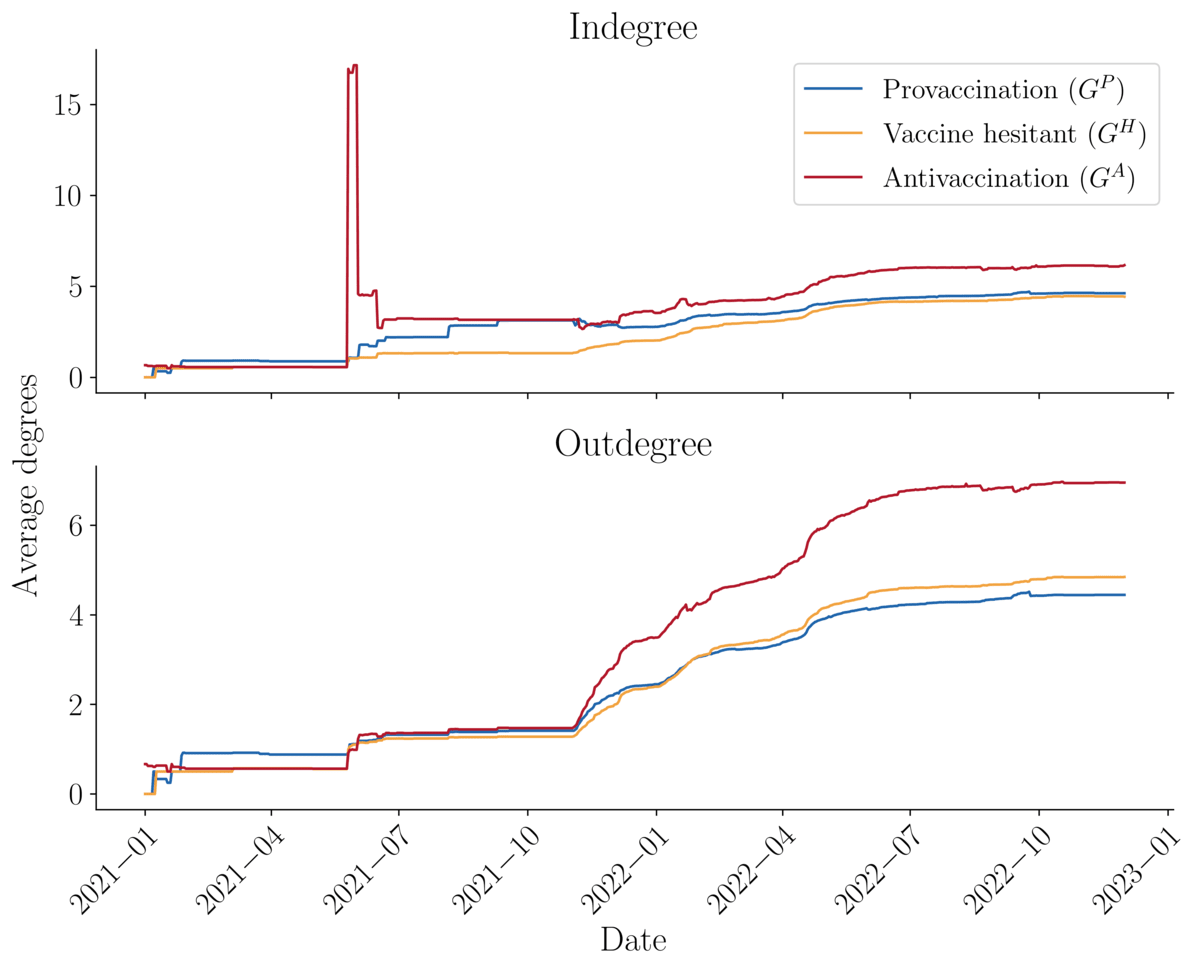
Average indegree and outdegree diversity by layer (temporal).

Temporally, these trends are generally consistent, with a few notable exceptions. Figure 5 tracks the average indegree and outdegree diversity by layer over time. Before November 2021, there is a notable peak in the average indegree for the antivaccination layer. This spike likely corresponds to a post that received significant attention, despite the antivaccination layer being relatively small at that time. Otherwise, the antivaccination layer maintains a higher average indegree consistently until December 2022. Regarding outdegree, all 3 layers show similar values until November 2021, after which the antivaccination layer exhibits a sharp increase followed by a gradual decline. This increase suggests intensified commenting behavior within the layer, potentially indicating a break in the echo chamber effect compared with the other 2 layers.

### Micro-Network Echo Chambering

The findings in the previous section indicate a push-and-pull effect between chambering and nonchambering dynamics. Although nodes common to both layers may exhibit some chambering tendencies, the antivaccination layer as a whole includes more nodes that extend across multiple layers. This section further explores the chambering trend by examining the contributions of 2 user types, high-diversity nodes and hardliners, within the multilayer network.

Comparing indegrees and outdegrees of highly diverse nodes across each layer reveals their polarized engagement, contributing to sentiment echo chambers. The results indicate that nodes with the most diverse participation predominantly receive and give comments on provaccination topics (Table 5). This trend is particularly substantial for indegrees, where most comments are received on the provaccination layer. For outdegree, there is no difference between the provaccination and hesitant layers, indicating that diverse nodes comment similarly on both topics and potentially disrupt the chambering effect. However, considering the larger size of the vaccine hesitant layer, this could imply that diverse nodes receive more comments from the provaccination layer, reinforcing chambering. Compared with the provaccination and hesitant layers, the antivaccination layer shows the least interaction from diverse nodes in terms of both giving and receiving comments. This trend is anticipated due to the smaller size of the antivaccination layer, and the proportion of connections aligns with its relative size compared with the other layers. These findings suggest that the forum as a whole may exhibit a preference for engaging with provaccination messaging, indicating a form of chambering, albeit in a protective manner. Diverse nodes are then spanners through the network.

**Table 5:** The proportion of connections into each vaccine topic layer by the top 50 diverse nodes (total).

Layer	*G* ^*P*^			*G* ^*H*^			*G* ^*A*^		
	Indegree, mean (CI)	Outdegree, mean (CI)	Indegree, mean (CI)		Outdegree, mean (CI)	Indegree, mean (CI)		Outdegree, mean (CI)	
Proportion of connections	54.63 (43.42-60.84)	44.40 (42.41-46.39)	33.07 (26.70-39.43)		40.51 (37.70-43.34)	12.30 (7.62-16.99)		15.09 (13.62-16.54)	

**Figure 6:**
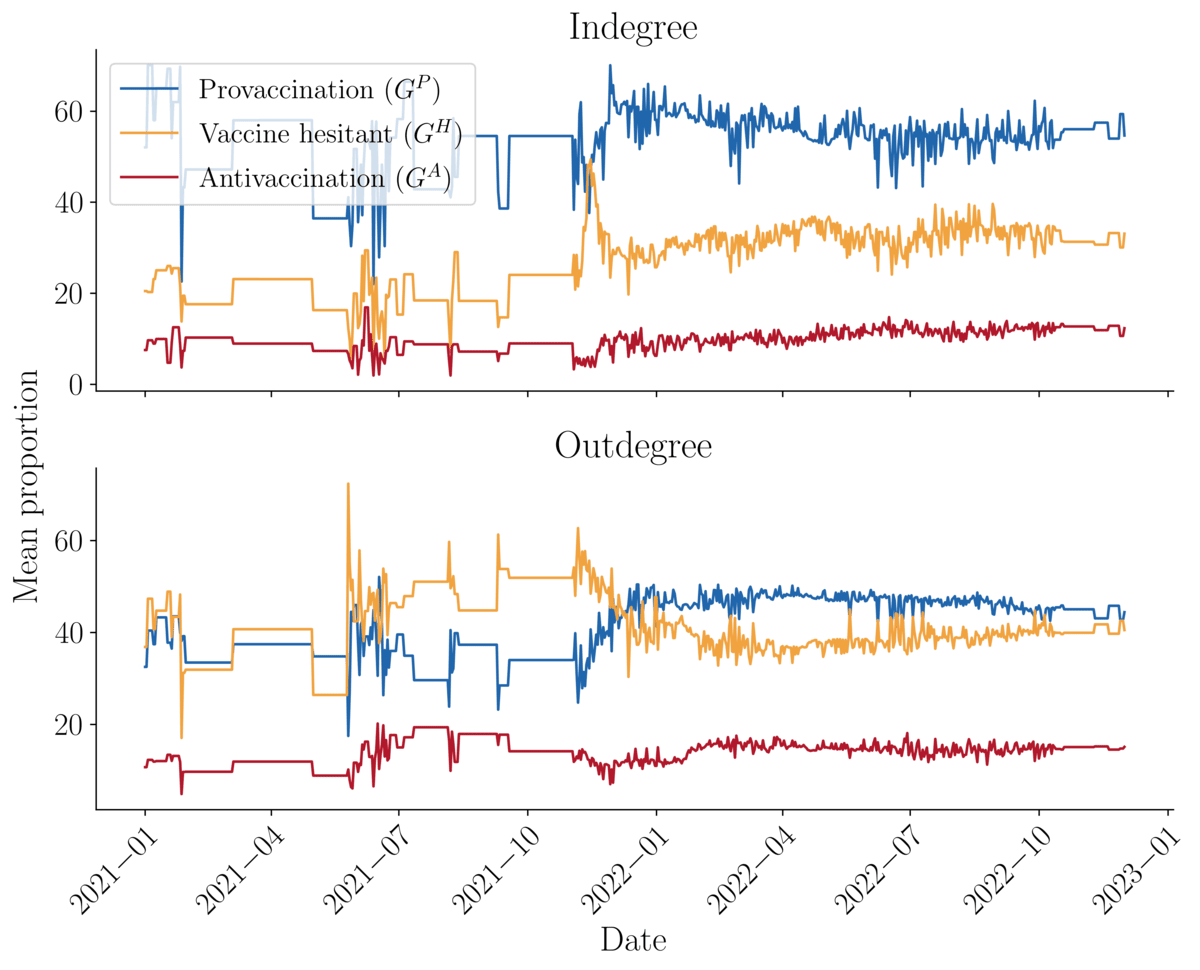
Proportion of connections into each vaccine topic layer by top-50 diverse nodes (temporal).

Temporally, these trends largely hold, with a notable exception in the outdegree for the vaccine hesitant layer (Figure 6). Initially, commenting from diverse nodes was predominantly directed at the vaccine hesitant layer, but by November 2021, there was a notable shift toward engagement with the provaccination layer. This shift may indicate a trend toward forming a “health community” facilitated by network spanners. Further reinforcing this point is that the gap between the provaccination and hesitant layers diminishes from July 2022 until the end of the period under study, indicating another shift in the chambering effect. This breaking of chambering is also evident in the indegree for the antivaccination layer, which shows a slight overall increase starting from November 2021. This suggests that the antivaccination layer is increasingly engaging with diverse nodes.

**Figure 7:**
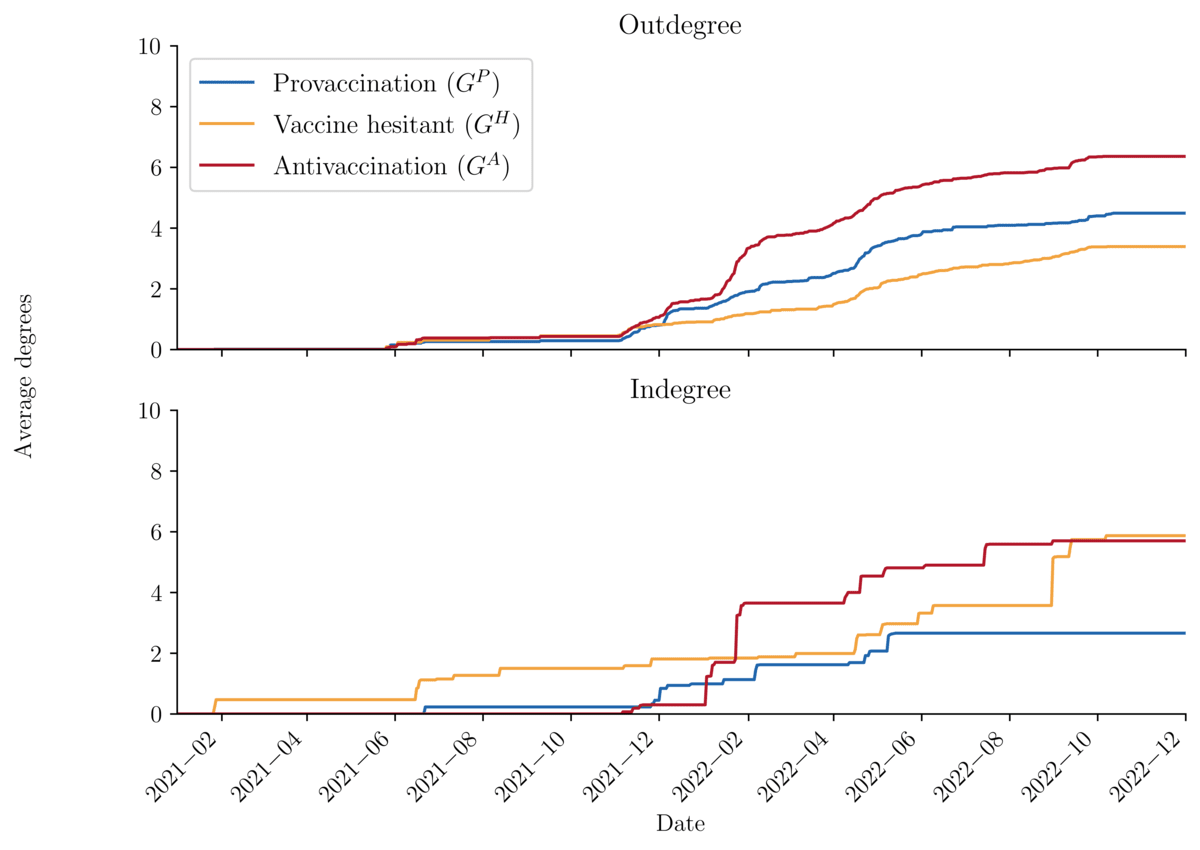
Temporal and cumulative outdegree and indegree growth among hardliners for each layer (temporal).

Hardliners (ie, those who predominantly engage within their own layer) are compared across layers to assess how they drive echo chambering within their respective layers. The results indicate that antivaccination hardliners are more active in commenting on antivaccination boards compared with the other 2 layers (Table 6). This trend remains consistent over time and shows a slight sharp increase in February 2022 during the Omicron outbreak in Taiwan (Figure 7, upper panel). However, this does not imply that the vaccine hesitant layer is entirely inactive within itself. The high indegrees suggest significant commenting within the layer, possibly indicating that a few nodes are responsible for disseminating information received by others. This trend gradually intensifies over time, with spikes observed during early vaccine procurement and the announcement of relaxed antiepidemic measures in late 2022 (Figure 7, bottom panel). Antivaccination hardliners predominantly receive information within their own group, particularly surging during the Omicron outbreak and continuing steadily thereafter. This engagement suggests that hardliners in the vaccine hesitant and antivaccination layers contribute to the entrenched ideologies within their respective groups over time.

**Table 6:** Total standardized outdegree and indegrees for hardliners within their own layer (total).

Layer	Outdegrees, mean (CI)	Indegrees, mean (CI)
*G^P^*	2.84 (2.81-2.88)	1.70 (1.62-1.77)
*G^H^*	2.15 (2.11-2.19)	3.71 (3.47-3.96)
*G^A^*	4.03 (3.97-4.10)	3.58 (3.38-3.78)

## Discussion

### Principal Findings

This study examined temporal chambering at the macro-network layer level and assessed the role of influential users in this process. Overall, the layers demonstrated chambering in their commenting behaviors, particularly evident in the strong polarization between the provaccination and anti-vaccination stances. Diverse nodes showed significant cross-layer engagement, particularly in the provaccination and vaccine hesitant layers. This contrasts with hardliners, who show more activity within the vaccine hesitant and antivaccination layers. These findings suggest strategies for information management moving forward and contribute to the evolving field of infodemic management.

Despite attempts to definitively assess the existence or absence of echo chambers, it is apparent that multiple chambering and nonchambering processes likely occur. For instance, analysis of similar nodes through Spearman ranks indicates varying degrees of chambering across layers and measurement criteria. The provaccination and antivaccination layers appear to exhibit the highest polarization on both measures, while the provaccination and vaccine hesitant layers show the least polarization. These findings align with previous network studies that have identified echo chambers in social media discussions on vaccines, particularly along the provaccine and antivaccine lines [24,26,29]. Additionally, the trend for receiving comments (indegree) shows less overall polarization compared with the trend in commenting (outdegree), suggesting that users may actively engage by expressing their own sentiment preferences.

Another significant factor contributing to chambering is the activity of specific nodes. Hardliners show significantly higher activity within the antivaccination layer compared with the other 2, indicating a stronger entrenchment effect (chambering). Strategies to mitigate this effect could include flagging or tagging posts from antivaccination hardliners [44]. Flagging represents an effort to balance the protection of an open, democratic forum while countering harmful messaging, akin to Singapore’s Protection from Online Falsehoods and Manipulation Act (POFMA) [45]. Additionally, involving moderators in content management [46,47] aimed at antivaccination hardliners could mitigate their impact on chambering [48]. Highly diverse nodes also allocate fewer comments proportionally to the antivaccination layer, with their engagement primarily centered around the provaccination layer, contributing to their diversity. These provaccination users can serve as valuable resources for disseminating promotional messages, given their demonstrated willingness to engage. Using these multipronged strategies concurrently aligns with common approaches to countering antivaccination movements [49].

Conversely, certain nodes engage in activities that disrupt chambering. The antivaccination layer exhibits the highest average indegree and outdegree compared with the other layers. This indicates that, apart from the nodes common to the Spearman calculations, a significant portion of this layer actively participates in both receiving and giving comments across the entire network. These findings contribute to the broader discourse on “structural hole spanners” within networks [50]. These individuals often act as bridges across diverse topics, engaging with users who hold cross-cutting attitudes toward vaccination [51]. These findings also support earlier research indicating that the vaccine hesitant group serves as a “battleground” where pro- and antivaccination narratives compete [25]. The intermediate Spearman rank values between the hesitant layer and the other 2 layers in both indegree and outdegree reinforce this idea, suggesting that the hesitant layer exhibits closer overlap with both groups.

Temporally, there are several active periods where trends shift. In the initial period from January to June 2021, the presence of a few boards makes each layer more responsive to new threads and comments, resulting in stepped patterns and high fluctuations. Surprisingly, despite Taiwan’s slow vaccine procurement and its first “large” outbreak (approximately 300 daily cases), which would typically attract more attention, the forum remains relatively silent on COVID-19 vaccine issues. Around June 2021, as vaccines become more readily available, several trends become more pronounced. The most significant changes occur in November 2021: cross-cutting users comment more on the provaccination layer, antivaccination hardliners become more insular, and polarization intensifies between the provaccine and antivaccine layers. During this period, various booster policies, the Delta outbreak, and the impending Omicron outbreak likely contributed to increased dialog around vaccines.

### Limitations and Next Steps

There are several limitations to this study and avenues for exploration, particularly in relation to the WHO’s infodemiology research agenda.

The first concern pertains to the data set. Social media platforms often attract specific user demographics and ideological stances. Additionally, the amount of data accessible through application programming interfaces may restrict the availability of scraped data, affecting the representativeness of the general population, a key goal in epidemiological studies. These inherent biases in social media data can internally skew the results. Despite PTT’s loose moderation and nonprofit nature, it is not immune to these limitations. To better contextualize the study, it is crucial to acknowledge its limitations stemming from these factors. To mitigate this bias, several approaches can be considered. First, conducting a comparative study—either on another topic within the same forum or across different platforms—would be beneficial. For instance, conducting similar analyses on DCard, an X-style platform known for attracting a younger demographic, could provide cross-validation of findings across different generations and platform types. This approach can also be extended to more international platforms such as X or Instagram. Another method is to validate the findings by comparing them with sentiment data from epidemiological sources. Conducting such comparative studies can enhance the reliability of these findings, ensuring that observed differences are not merely artifacts of platform or network size variations. These methods align well with the infodemic research agenda, emphasizing the triangulation of diverse data types to better integrate epidemiological and infodemiology insights.

Another limitation is that the definition of “influence” is not exhaustive in this study. The study identifies influential users using indicators such as diversity and hardliners, but these metrics are not comprehensive. The choice of indicators is often constrained by the platform’s structure and available data. For instance, follower count could be a measure of influence on platforms such as X, but PTT lacks such indicators. Despite these limitations specific to PTT, a broader array of metrics for measuring influence remains possible. Indeed, measuring the percentage of positive or negative engagement based on sentiment markers of user posts, as well as their active time spent on the forum, could provide additional dimensions to assess influence. Exploring a spectrum of influence metrics across comparative studies could further enrich our understanding of user dynamics within different platforms.

To better gauge the actual impact of “influential users” on chambering and network structure, conducting network simulations could be highly beneficial. These simulations could help bridge the gap between identifying influential users based on various metrics and understanding how their behaviors contribute to the overall network dynamics and chambering phenomena. Exploring these methods would indeed advance the understanding of chambering dynamics in online discussions about vaccination. Conducting network simulations where influential nodes are systematically removed can reveal how their presence or absence affects the clustering and cohesion of different sentiment layers. Additionally, performing regressions at the user level to analyze factors influencing their engagement across provaccine, vaccine hesitant, and antivaccine stances could provide insights into the drivers of polarization and chambering within the network. These approaches align well with the goals of infodemic management during epidemics, as outlined in the WHO research agenda.

Understanding the underlying reasons behind chambering trends in online discussions about vaccination is crucial for preparing and responding to future infodemics effectively. Identifying whether these trends are triggered by outbreaks, policy changes, or other factors unrelated to outbreaks can provide insights into the temporal dynamics of chambering. This knowledge aids in tracking infodemics and vaccine sentiment on social media platforms. Integrating this narrative with analyses of clustering and network dynamics helps illustrate the cause-effect chains driving chambering and their broader impacts on public discourse and health communication strategies. This comprehensive distillation of pathways represents a critical step in the broader paradigm shift toward integrating epidemic studies with infodemic studies, rather than treating them as separate entities.

The importance of overlapping across the layers is underemphasized in this study and carries implications for construct validity. The current framework assumes that each layer encapsulates all sentiments related to a specific vaccine stance, essentially forming a “discussion sphere” or “space” around that stance. Users can comment in multiple layers because they hold diverse vaccine sentiments, thereby blurring the exclusivity of layers and indicating cross-cutting behavior, which breaks the chambering effect. A clearer illustration of how users comment across layers over time would help distinguish true chambering from typical forum behavior where users engage across multiple boards.

The final major limitation is the absence of content analysis. Although layer- and node-level metrics serve as proxies for cross-layer communication, the specific direction and nature of engagement, such as the content exchanged, are unclear without detailed content analysis. Understanding the content aspect would provide further insights into how engagement occurs between these layers and users. This also partially addresses the issue of overlapping, shifting from engagement across layers as a measure of chambering to engagement with similar vaccine sentiments. Further along the infodemic management agenda, understanding content also informs how interventions should be designed. For instance, engagement with pro- and antivaccination content could lead to either constructive or destructive discussions. Focusing on specific content types, such as misinformation, further illustrates the existence or formation of echo chambers. This approach also aligns with the WHO’s research agenda on infodemics, linking echo chambering to unhealthy behaviors and potentially serving as a focal point for public health interventions.

## Abbreviations

BBS: bulletin board system
CDC: Centers for Disease Control and Prevention
POFMA: Protection from Online Falsehoods and Manipulation Act
WHO: World Health Organization

## Data Availability

Data from PTT are publicly available. Data used for this analysis are available upon reasonable requests made to the author of this paper.
