# Correction: Vaccine Hesitancy in Taiwan: Temporal, Multilayer Network Study of Echo Chambers Shaped by Influential Users

**DOI:** 10.2196/65413

**Published:** 2024-08-15

**Authors:** Jason Dean-Chen Yin

**Affiliations:** 1 School of Public Health Li Ka Shing Faculty of Medicine University of Hong Kong Hong Kong China (Hong Kong)

In “Vaccine Hesitancy in Taiwan: Temporal, Multilayer Network Study of Echo Chambers Shaped by Influential Users” (Online J Public Health Inform 2024;16:e55104) the following information has been added:

The “Ethical Considerations” section has been changed to read as follows:

All data from PTT are open and publicly available. All data from PTT in its raw form include usernames. These usernames are deidentified and anonymised during the research process to ensure they cannot be traced back to individuals. To do this, a 6 digit alphanumeric temporary username is generated for each unique user. Data is available upon request.

Figure 1 will now appear as the following image, with updated caption:

Figure 1. Chinese and English labelling of 1 thread of the PTT forum to show its structure. Usernames omitted for protecting individual identity.

**Figure 1:**
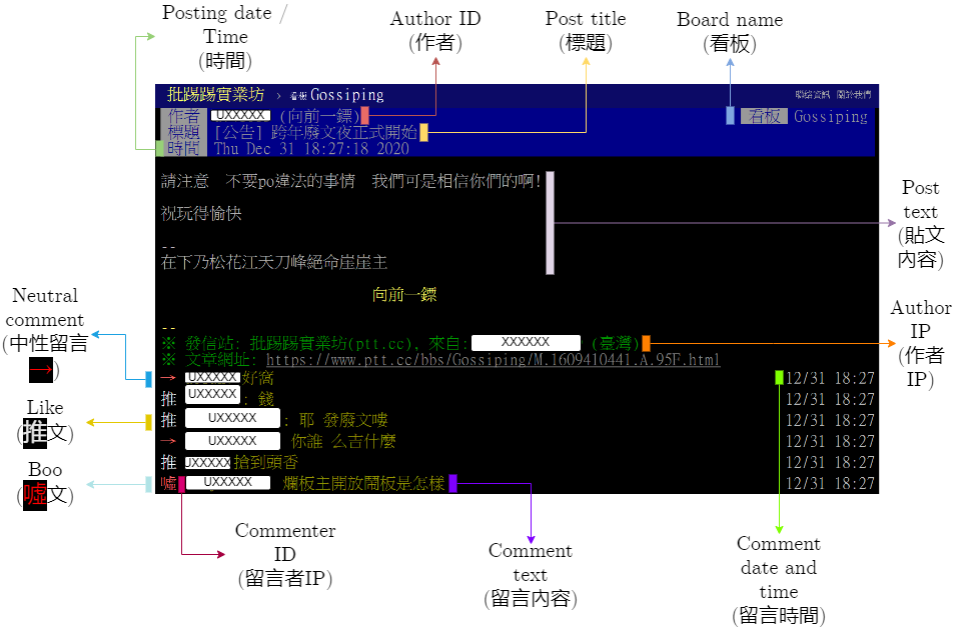
Chinese and English labelling of 1 thread of the PTT forum to show its structure. Usernames omitted for protecting individual identity.

The Data Availability statement has been amended to read as follows:

Data from PTT are publicly available. Data used for this analysis are available upon reasonable requests made to the author of this paper.

Finally, the file previously present as Multimedia Appendix 1 was removed.

The correction will appear in the online version of the paper on the JMIR Publications website on August 15, 2024, together with the publication of this correction notice. Because this was made after submission to PubMed, PubMed Central, and other full-text repositories, the corrected article has also been resubmitted to those repositories.

